# Sensitivity of stratospheric ozone to the latitude, season, and halogen content of a contemporary explosive volcanic eruption

**DOI:** 10.1038/s41598-023-32574-9

**Published:** 2023-04-20

**Authors:** Freja F. Østerstrøm, J. Eric Klobas, Robert P. Kennedy, Anita Cadoux, David M. Wilmouth

**Affiliations:** 1 Harvard John A. Paulson School of Engineering and Applied Sciences, Harvard University, Cambridge, MA USA; 2 Department of Chemistry, University of Copenhagen, Copenhagen, Denmark

**Keywords:** Atmospheric chemistry, Atmospheric chemistry, Atmospheric chemistry, Atmospheric chemistry, Atmospheric chemistry

## Abstract

We present a systematic evaluation of the perturbation to the stratosphere from an explosive volcanic eruption injecting sulfur dioxide into the atmosphere, as a function of latitude, season, and injection gas halogen content in a chemistry-climate state representative of the present day (modeled as year 2025). Enhancements in aerosol surface area density and decreases in stratospheric ozone are observed for a period of years following all modeled scenarios, with volcanic eruptions near the equator impacting both hemispheres relatively equally, and eruptions at higher latitudes reducing the thickness of the ozone layer more substantially in the hemisphere of the eruption. Our simulations reveal that there that are significant seasonal differences when comparing the stratospheric impact of a volcanic eruption occurring in summer versus winter, and this holds true regardless of whether volcanic halogen gases (Cl, Br) are co-injected with sulfur dioxide. If an explosive halogen-rich eruption were to occur, there would be substantial ozone losses in both hemispheres, regardless of latitude or season, with recovery potentially exceeding 4 years.

## Introduction

Large explosive volcanic eruptions can result in the injection of tremendous quantities of trace gases into the stratosphere. Water vapor is the trace gas with the highest number density in all volcanic eruption columns, originating from the magma itself, from entrainment of ambient air, or from the vaporization of surface water. The recent eruption in January 2022 of the Hunga Tonga-Hunga Ha’apai submarine volcano (20.536$^{\circ }$S, 175.382$^{\circ }$W) injected an unprecedented amount of water vapor (up to 10% of the stratospheric water content^1,2^) high into the stratosphere. Despite the absolute importance of water vapor in controlling the speciation and transport of other trace gases within the eruption column, sulfur dioxide (SO$_\text {2}$) is frequently considered the most impactful of the volcanic gases due to its role in ozone depletion and its influence on global climate. Enhancements in sulfate aerosol following the injection and subsequent oxidation of SO$_\text {2}$ increase the upward scattering of shortwave radiation back to space, effectively reducing the incident shortwave-induced heating of the surface.

**Figure 1:**
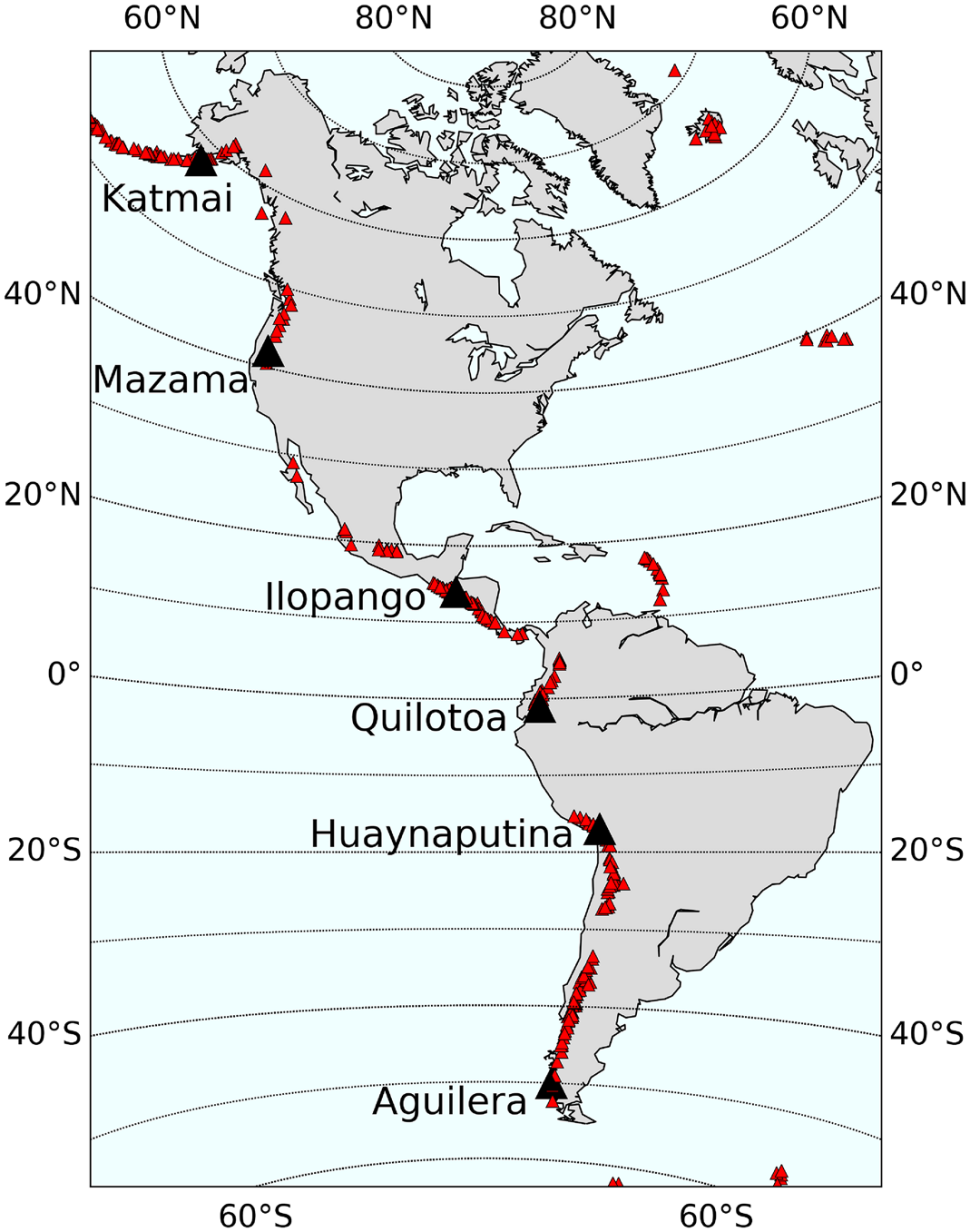
Locations of 314 stratovolcanoes known to have erupted in the western hemisphere during the Holocene era (red triangles). The coordinates of six of these volcanoes (black triangles), whose Holocene history is known to have been marked by large explosive eruptions with Volcanic Explosivity Index (VEI) 6 or greater, were selected for investigation in this work. The map was generated using the Matplotlib Basemap Toolkit v1.1.0: https://matplotlib.org/basemap/^76^.

**Figure 2:**
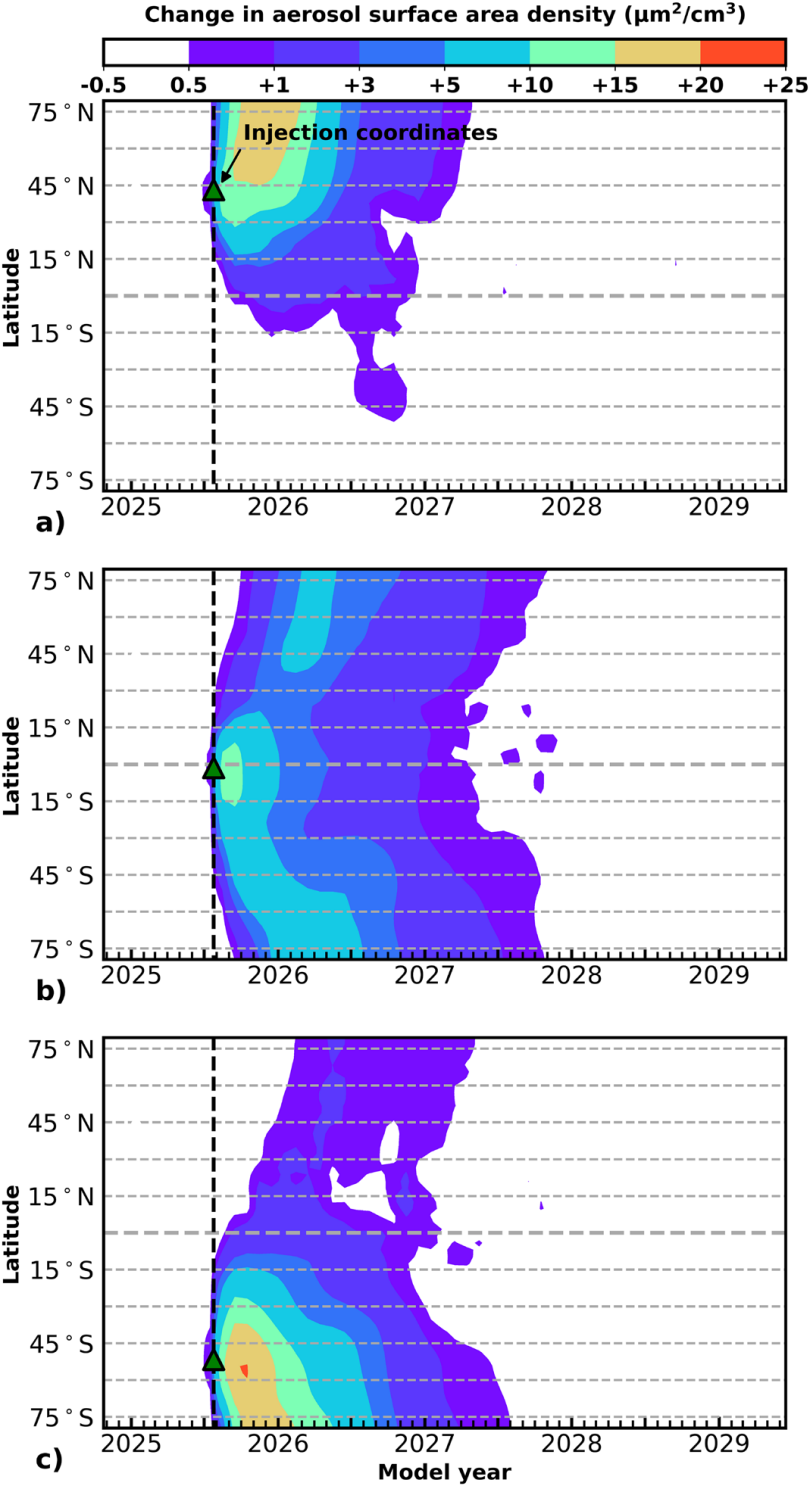
Aerosol surface area density (SAD) response to 5 Tg SO$_\text {2}$ injection (SSP3-70 July 25, 2025) as a function of latitude. In all panels, computed metrics reflect the difference in SAD between the volcanic eruption perturbation model ensemble and the baseline control model ensemble shown for a 4-year time-horizon. Total column response of aerosol SAD is indicated by the colorbar. The latitudes of eruption are: (**a**) 42$^\circ$N, (**b**), 1$^\circ$S and (**c**) 50$^\circ$S. Latitude and date of each SO$_\text {2}$ injection are indicated by a green triangle and a dashed line.

Sulfate injections by tropical, large, explosive volcanic eruptions can significantly perturb stratospheric ozone, as was observed following the Plinian eruption of Mt. Pinatubo (Philippines) in 1991 injecting 14–23 Tg SO$_\text {2}$ into the stratosphere^3–7^. The eruption plume contained halogen species corresponding to a HCl:SO$_\text {2}$ ratio of approximately 0.4, but perhaps due, in part, to the transit of the tropical typhoon Yunya directly over the plume, all inorganic halogen species were essentially removed in the wet troposphere before reaching the stratosphere^8–10^. While the coincident typhoon produced a significant increase in water vapor, transport of halogen species to the stratosphere is limited by multiple factors, such as heterogeneous reactions and removal by wet deposition; see Textor et al.^11^ for a detailed description of the microphysics of volcanic eruption columns. Though hydrogen halides can represent a significant fraction of the trace volcanic gases, they are highly water soluble. It was believed until recently, on the basis of the limited observational record and microphysical-thermodynamic eruption column modeling, that hydrometeor scavenging efficiently scrubs the entirety of these halogen species from the eruption column prior to injection into the stratosphere^12^. However, the studies from which this conclusion was derived neglected important processes, such as sophisticated aerosol salinity microphysics or aqueous interhalogen chemical cycling, which may increase the fraction of halogens injected above the tropopause^11,13,14^.

During the two years following the Mt. Pinatubo eruption, stratospheric ozone losses up to or exceeding 10% were reported in the northern mid-latitudes^15–17^ as well as stratospheric temperature increases of up to 3 K^15,18^, depending on the time horizon of analysis. These reductions in column ozone thickness occurred primarily as a consequence of changes in the rates of certain heterogeneous chemical reactions following the orders-of-magnitude enhancement in the stratospheric aerosol burden^19,20^. Local heating effects from the aerosol disrupted stratospheric circulation, effectively reducing transport of ozone from tropical regions to the northern mid-latitudes and producing enhancements in ozone layer thickness in the Southern Hemisphere^5,21,22^. Even though the Mt. Pinatubo eruption did not measurably enhance the concentration of halogens in the stratosphere, the sulfate aerosol cloud changed the rates and conditions controlling heterogeneous chemistry^5,17,23–25^, leading to changes in inorganic halogen partitioning. This effect on ozone response to a volcanic eruption is also dependent on the background levels of available halogen species and the halogen response to the chemistry-climate state of the atmosphere (quantified by Equivalent Effective Stratospheric Chlorine (EESC) and Equivalent Effective Stratospheric Benchmark-normalized Chlorine (EESBnC))^26–28^.

Geochemical analyses of volcanic glasses (i.e., quenched magmatic liquids) and ice core analyses indicate that some pre-industrial, large-scale (volcanic explosivity index, VEI, 6–7) eruptions released tens to several hundred Tg of Cl (e.g., the 1815 eruption of Tambora in Indonesia; the 1613 BCE Minoan eruption in Santorini, Greece; the 1257 eruption of Mount Samalas in Indonesia)^29–33^, far exceeding the total annual Cl flux from satellite-era volcanism of approximately 5 Tg^34^. If even <2% of emissions from these halogen-rich eruptions were to survive transport to the contemporary stratosphere, profound reductions in total column ozone would result, as was demonstrated in model simulations of similar halogen injections by Klobas et al. in contemporary and future chemistry-climate scenarios^35^ and in evaluations of eruptions in paleoatmospheric states^31,33,36–39^. No recent volcanic eruptions have injected large amounts of HCl into the stratosphere.

Large, explosive, high-latitude volcanic eruptions likely pose less of a threat to global human health than eruptions at other latitudes because, during these events, sulfate aerosol is injected into subsiding air masses, with correspondingly lower aerosol burden e-folding decay times than found with tropical eruptions^40^. It follows that the lower global stratospheric aerosol enhancement would result in smaller perturbations of heterogeneous chemical reactions and correspondingly reduced impacts on global stratospheric ozone. Because the magnitude and extent of subsidence of stratospheric air at high latitudes is a strong function of season, with downwelling maximizing in the winter when shortwave heating of the stratosphere is minimal, the ozone impact of a high-latitude eruption may be a function of seasonality similar to its climate impact^40–43^, but it has not been evidenced to date.

**Figure 3:**
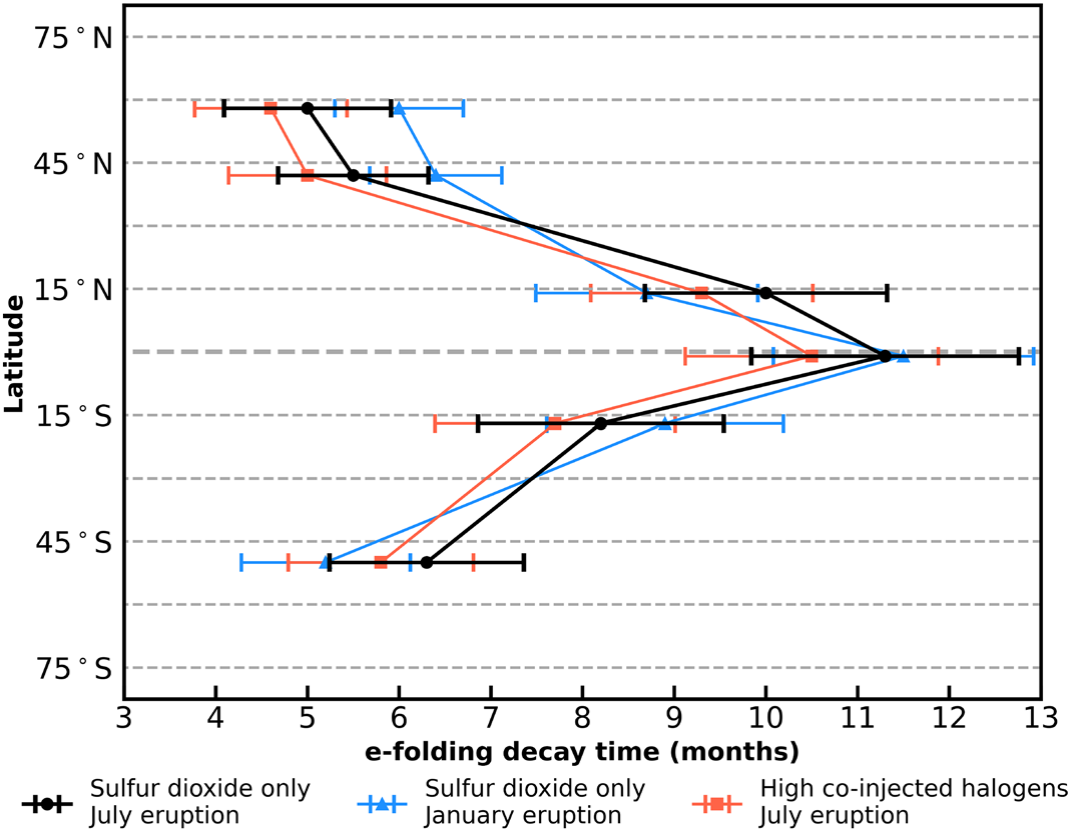
Average global sulfate aerosol e-folding decay time as a function of eruption latitude (SSP3-70 January/July 2025). Volcanic eruptions in January (blue) and July (black and red) 2025. The symbols indicate the volcanic stratospheric injection and season: 5 Tg SO$_\text {2}$ (sulfur dioxide only, black circles and blue triangles) and 5 Tg SO$_\text {2}$, 0.5 Tg HCl, 0.005 Tg HBr (high co-injected halogens, red squares). The bars indicate the 2$\sigma$ variation of the model ensembles.

**Figure 4:**
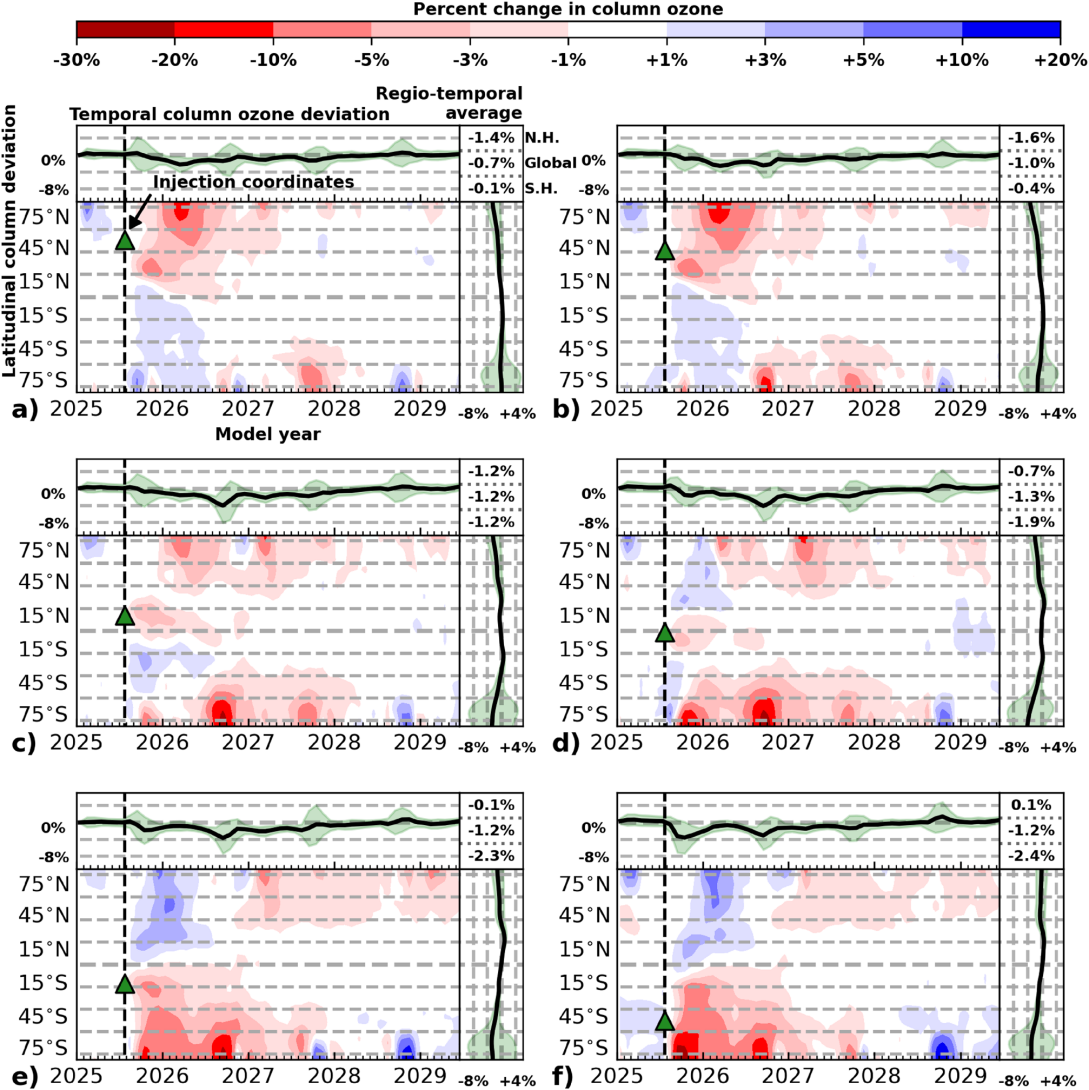
Ozone response to 5 Tg SO$_\text {2}$ injection (SSP3-70 July 25, 2025) as a function of latitude. The latitudes of eruption for panels (**a**) through (**f**) are: 58$^\circ$N, 42$^\circ$N, 14$^\circ$N, 1$^\circ$S, 17$^\circ$S, and 50$^\circ$S, respectively, with the latitude and date of SO$_\text {2}$ injection indicated by a green triangle and a dashed line in each panel. In all graphical panels, computed metrics reflect the percent change in ozone between the volcanic eruption perturbation model ensemble and the baseline control ensemble shown for a 4-year time-horizon. Main panels (bottom left): Response of total column ozone in percent to the volcanic eruption as indicated in the colorbar (note that colorbar levels increment non-linearly). Top left panels: Global average response of total column ozone over time as indicated in the left scale. Bottom right panels: Response of total column ozone average over time versus latitude. Green shading in the top left and bottom right panels illustrates the variation (2$\sigma$) in the eruption perturbation ensemble; note that the increment between dashed lines in these panels is 4%. Top right corner panel: 3-year extra-polar hemispherical average (0$^\circ$–80$^\circ$N and 0$^\circ$–80$^\circ$S) and global average (80$^\circ$S–80$^\circ$N) of the total column ozone deviation, ordered as NH, Global, SH.

To our knowledge, there has been no prior systematic exploration of the impact of the latitude or the season of a volcanic eruption on total column ozone. While several recent studies have investigated the impact of halogens co-injected with SO$_\text {2}$ from mostly historic volcanic eruptions^31,39,44–46^, no previous study has examined the impact of halogen co-injection as a function of latitude and season. Given the critical importance of stratospheric ozone in protecting life on this planet, this study was undertaken to improve our understanding of a variety of realistic, modern-day volcanic eruption scenarios and better constrain the risks and uncertainties. We use 30-member perturbed initial conditions ensembles of experiments to evaluate the impact of latitude, seasonality, and halogen content of large, explosive volcanic eruptions on the stratosphere.

**Figure 5:**
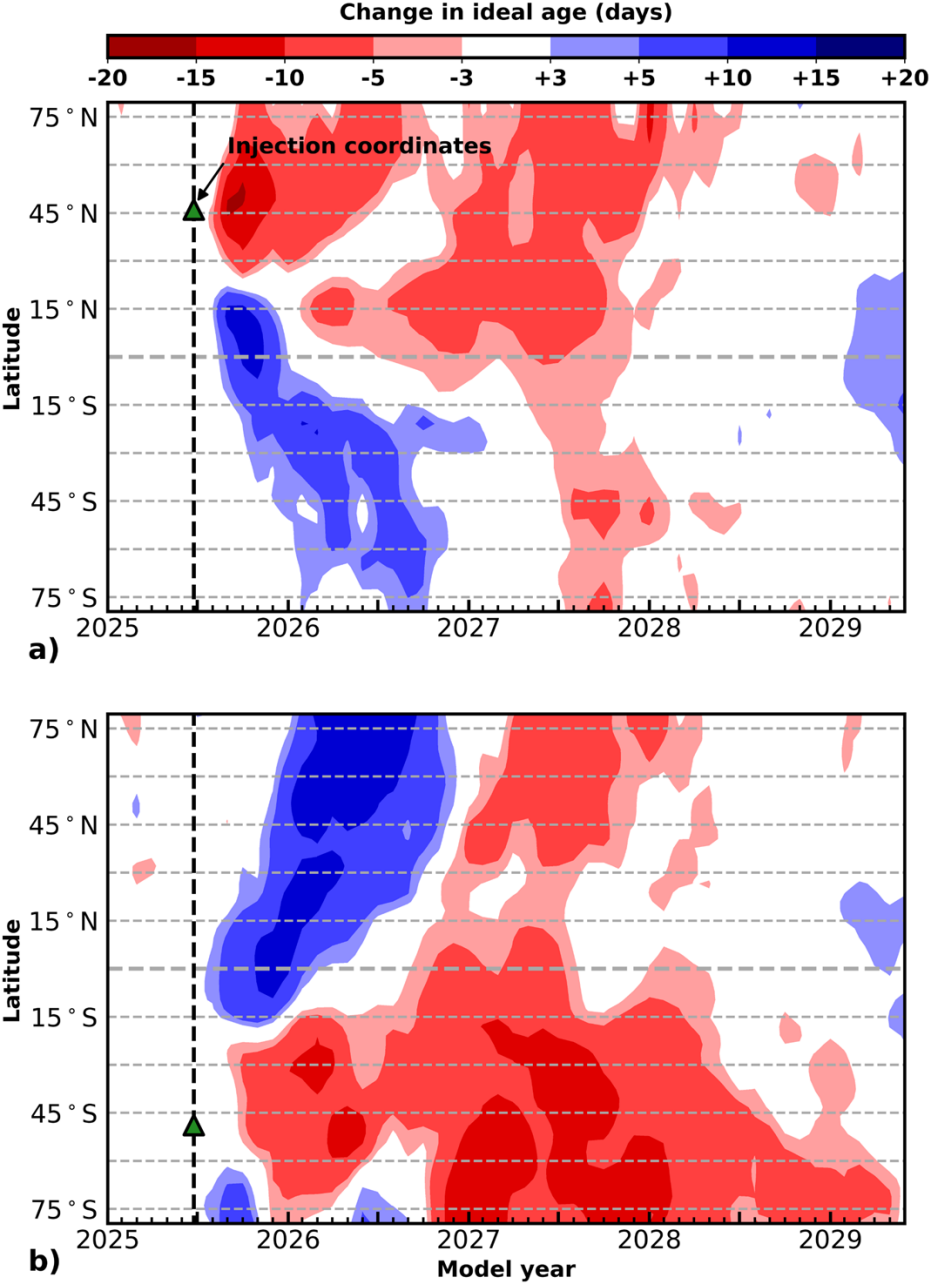
Ideal age of air response to 5 Tg SO$_\text {2}$ injection (SSP3-70 July 25, 2025) as a function of latitude and time, where ideal age is a modeled variable that describes the age of air relative to its transport from the surface. In both panels, computed metrics reflect the difference between the eruption perturbation ensemble and the control ensemble with a 4-year time-horizon, and the latitude and date of SO$_\text {2}$ injection is indicated by a green triangle and a dashed line. The latitudes of eruption are 42$^\circ$N and 50$^\circ$S for panels (**a**) and (**b**), respectively.

**Figure 6:**
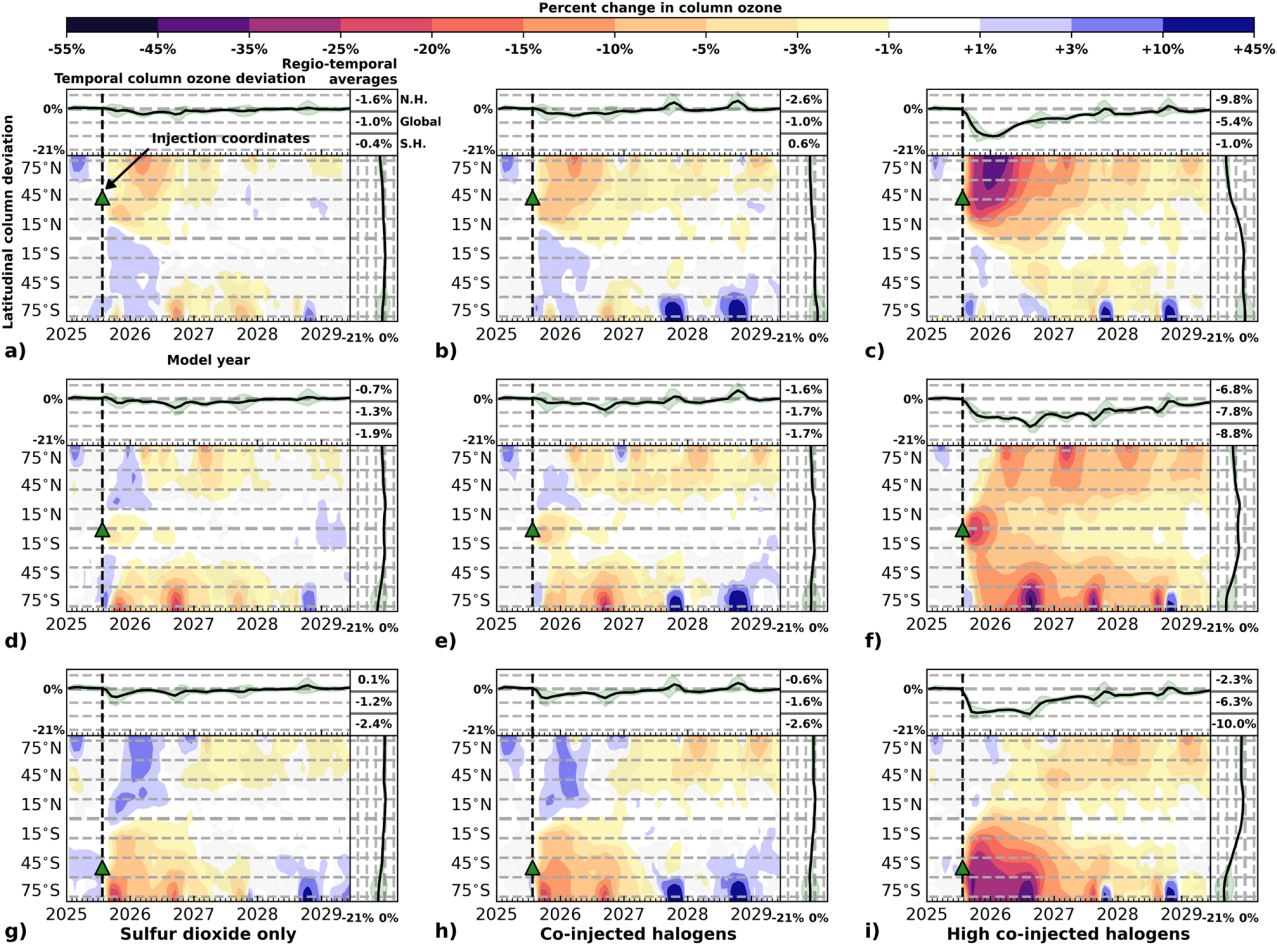
Ozone response to volcanic eruptions of varying halogen injection. Left column, panels (**a**), (**d**), and (**g**): Sulfur dioxide only eruptions in which volcanic SO$_{\textrm{2}}$ (5 Tg SO$_\text {2}$) is injected into the stratosphere. Center column, panels (**b**), (**e**), and (**h**): Intermediate co-injected halogen eruptions in which 5 Tg SO$_\text {2}$, 0.05 Tg HCl, and 0.0005 Tg HBr are injected into the stratosphere. Right column, panels (**c**), (**f**), and (**i**): High co-injected halogen eruptions in which 5 Tg SO$_\text {2}$, 0.5 Tg HCl, and 0.005 Tg HBr are injected into the stratosphere. Volcanic eruption scenarios (SSP3-70 July 25, 2025) are presented for three latitudes: (**a**)–(**c**) 42$^\circ$N, (**d**)–(**f**) 1$^\circ$S, and (**g**)–(**i**) 50$^\circ$S. In all graphical panels, computed metrics reflect the difference between the eruption perturbation ensemble and the control ensemble with a 4-year time-horizon. Main, bottom left panels: Response of total column ozone in percent as indicated in the colorbar (note that colorbar levels increment non-linearly). Top left panels: Global average response of total column ozone over time as indicated in the left scale. Bottom right panels: Response of total column ozone average over time versus latitude. Green shading in the top left and bottom right panels illustrates variation (2$\sigma$) in the eruption perturbation ensemble; note that the increment between dashed lines in these panels is 7%. Top right corner panel: 3-year extra-polar hemispherical average (0$^\circ$–80$^\circ$N and 0$^\circ$–80$^\circ$S) and global average (80$^\circ$S–80$^\circ$N) of the total column ozone deviation, ordered as NH, Global, SH. Panels (**a**)–(**i**): Latitude and date of SO$_\text {2}$ and halogen injection are indicated by a green triangle and a dashed line.

## Methods

Model evaluations were conducted with the SOCOL-AERv2 3D chemistry-climate-aerosol model as described in Feinberg et al.^47^ with T42 spectral grid resolution (2.8$^\circ$ x 2.8$^\circ$ at the equator) and 39 pressure levels spanning the surface to 0.1 hPa. SOCOL-AERv2 is a chemistry-climate-aerosol model with detailed sectional aerosol microphysics derived from the AER-2D model^48–50^. The SOCOL-AER, SOCOL-AERv2, and AER-2D models have been used extensively in the past for evaluations of volcanic aerosol evolution and consequential changes in the stratospheric trace gas inventory due to enhancements in heterogeneous chemical reactions^16,35,51–55^. The implementation of SOCOL-AERv2 employed in this work is identical to the model described in Feinberg et al.^47^ except for the following modifications: (1) Chemical boundary condition emissions were harmonized with prescribed Shared Socioeconomic Pathways (SSP) scenarios boundary conditions including aircraft emissions of carbon monoxide, non-methane volatile organic compounds, SO$_\text {2}$, and surface emissions of dimethyl sulfide. (2) Heat fluxes for forcing a mixed-layer ocean were computed on a monthly, transient basis from a 10-member perturbed initial conditions ensemble. For each SSP condition, initial month CO$_\text {2}$ boundary conditions were perturbed by a unique random value between $\pm 1$% of the prescribed value. Sea surface temperatures and sea ice thickness were prescribed to MIROC6^56^ and obtained from the CMIP6 database. Each ensemble member was spun up with 20 years of time-slice conditions corresponding to the year 2015. Following spin-up, members were computed with transient SSP boundary conditions from the year 2015 until the year 2100. Climatological heat fluxes were then computed from the ensemble averages for each year of simulation.

For each experimental condition, non-volcanic control statistics were obtained using 30-member perturbed initial conditions ensembles, in which initial month CO$_\text {2}$ boundary conditions were modulated by a unique random value between $\pm 1$% of the prescribed value. The ensemble members were then allowed to evolve with transient boundary conditions for seven years. Because each ensemble condition resulted in 2.1 TB of data, monthly ensemble statistics were computed prior to data analysis, reducing data requirements to 96 GB for each evaluated condition. Likewise, each volcanic eruption condition was evaluated in 30-member perturbed initial conditions ensembles, and ensemble statistics were computed and stored for analysis in the same manner as the non-volcanic control conditions. This study included a total of 37 ensembles, comprised of 1110 individual model evaluations, totalling 7770 model years with a combined wall-clock run-time of about 6 years. Volcanic eruption scenarios were initiated in the stratosphere with an injection between 113.3 hPa and 13.5 hPa (about 15–30 km altitude dependent on location) of 5 Tg SO$_\text {2}$ according to the vertical profile prescribed by the R001-3D simulation of Sheng et al.^51^, in which SOCOL-AER was used to tune the stratospheric vertical mass injection profile of SO$_\text {2}$ in order to optimize the match between spatiotemporal evolution of the Mt. Pinatubo volcanic aerosol veil and satellite observation.

Halogen-rich eruption simulations were initiated by including co-injections of HCl and HBr with SO$_\text {2}$ using masses of the halogen species scaled to the same mass vertical profiles as used for the injection of SO$_\text {2}$ only. The upper bound for halogen injection masses was based on petrological data obtained from the 7.7 kya eruption of Mt. Mazama (42$^\circ$N, Oregon, USA) with an HCl:SO$_\text {2}$ ratio of 0.1^57,58^. The lower bound was chosen as an order of magnitude smaller HCl:SO$_\text {2}$ ratio of 0.01, an assumption that is consistent with satellite-era eruptions between 2005–2014 reported in Carn et al.^59^ with a range of HCl:SO$_\text {2}$ ratios being 0.01–0.03. The data were scaled to a stratospheric injection magnitude of 5 Tg SO$_\text {2}$. The stratospheric injection quantities are 0.05 Tg HCl and 0.0005 Tg HBr for the intermediate co-injected halogen scenario and 0.5 Tg HCl and 0.005 Tg HBr for the high co-injected halogen scenario, corresponding to HCl:SO$_\text {2}$ ratios of 0.01 and 0.1, respectively, and a HBr:HCl ratio of 1:100, which is representative of the natural abundances of the halogens in subduction and high SiO$_\text {2}$ rhyolites^60^. These scenarios can represent different-sized explosive eruptions (with a magnitude typical of VEI 5–6) with varying stratospheric transport efficiency of SO$_\text {2}$ and halogen content from the eruption column. Note that the 7.7 kya (VEI 7) eruption of Mt. Mazama is estimated to have released 217 Tg Cl in total^61^, which is comparable to the Cl output of other very large eruptions, such as the 1257 Samalas eruption (Indonesia) or the so-called Minoan ($\approx$3.6 kya) Santorini eruption (Greece)^31,32^. If 0.05 Tg HCl reached the stratosphere following the Mt. Mazama eruption (as in the intermediate co-injected halogen scenario), it would represent 0.02% of the total Cl output from the eruption reaching the stratosphere. The 0.5 Tg HCl in the high co-injected scenario would represent 0.2% of the total Cl reaching the stratosphere. These values (0.02–0.2%) are far less than the estimate of 3.7% given by Zdanowicz et al.^58^, who calculated a maximum stratospheric loading of 8.1 Tg Cl from the Mt. Mazama eruption based on their study of the Greenland Ice Sheet Project II core. The combined chlorine and bromine injections in the halogen co-injection scenarios in this study result in an enhancement in EESC of approximately 70 or 700 pptv relative to an EESC background of 1650 pptv for 2025 (3-year mean age-of-air), for the intermediate and high halogen co-injection scenario, respectively, using a time-dependent bromine alpha factor of 70^28^. The halogen enhancements will be larger and more dramatic in the regions closer to the location of the eruption.

**Figure 7:**
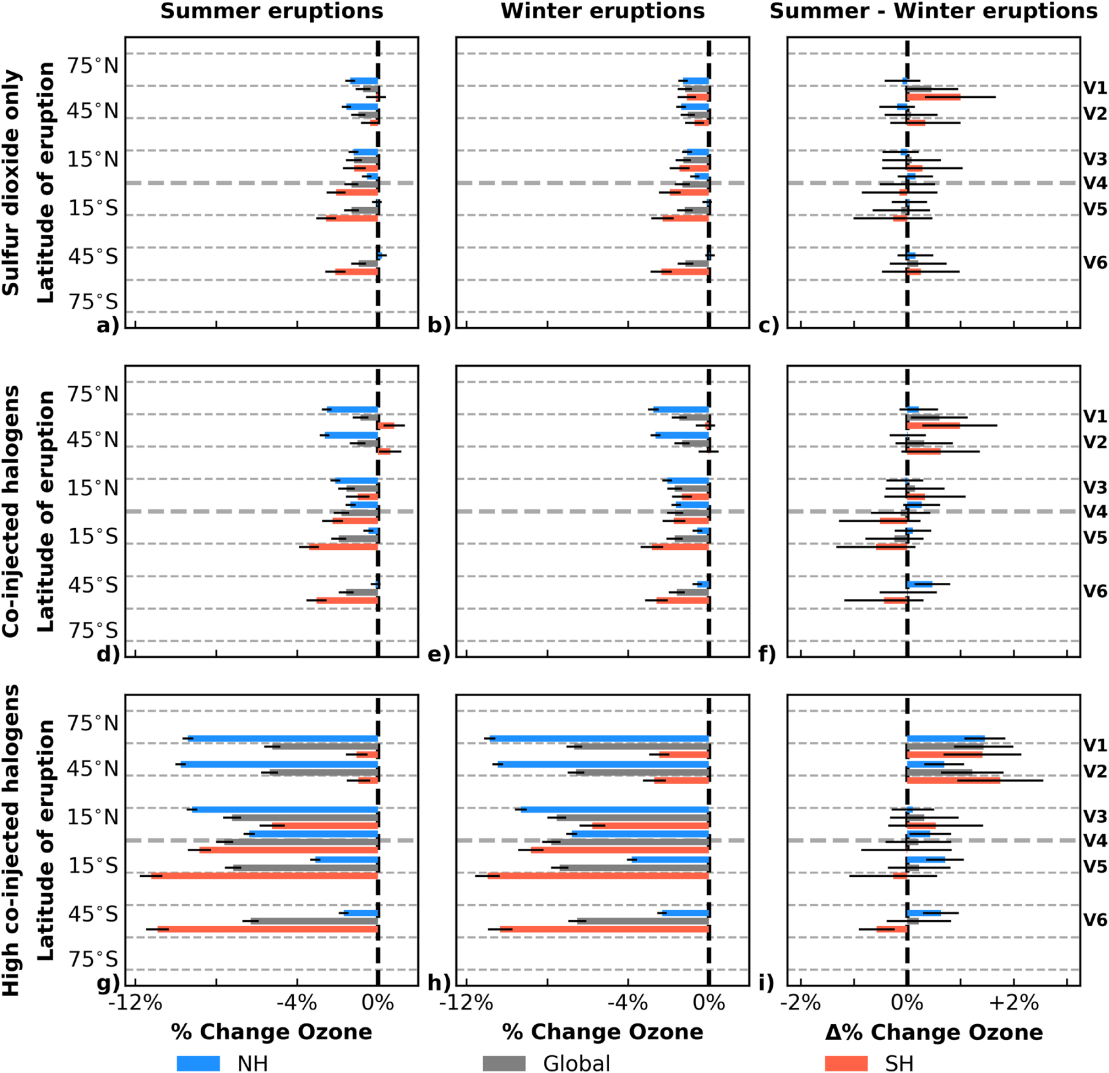
Ozone response dependence on seasonality of a volcanic eruption (SSP3-70 January/July 2025). Global (grey) and hemispherical (NH: blue, SH: red) changes in percent ozone over 3 years after a volcanic eruption shown for summer (left column) and winter (middle column) eruptions. The difference of summer–winter is shown in the right column. Panels (**a**)–(**c**) show the sulfur dioxide only eruptions, (**d**)–(**f**) the intermediate co-injected halogen eruptions, and (**g**)–(**i**) the high co-injected halogen eruptions. Volcanic eruption scenarios are divided into seasons for the NH and SH, with January representing NH winter and SH summer and July representing NH summer and SH winter. Within each panel, V1–V6 indicates eruption latitude: 58$^\circ$N, 42$^\circ$N, 14$^\circ$N, 1$^\circ$S, 17$^\circ$S, and 50$^\circ$S, respectively. Uncertainty whiskers are plotted in black, representing 1$\sigma$.

**Table 1:** Percent change in ozone across the globe, 5 months after the eruption.

		January 25, 2025 eruption			July 25, 2025 eruption		
Latitude of eruption	Latitude	Sulfur dioxide only	Intermediate co-injected halogens	High co-injected halogens	Sulfur dioxide only	Intermediate co-injected halogens	High co-injected halogens
58$^\circ$N	60$^\circ$N	−7.3	−11	−33	−3.2	−7.2	−39
	30$^\circ$N	−4.9	−7.4	−22	−4.9	−7.2	−23
	0$^\circ$	+0.3	−0.3	−6.5	+1.4	+1.3	−2.4
	30$^\circ$S	+5.0	+4.7	+0.5	+2.3	+1.9	+1.0
	60$^\circ$S	−0.8	−0.2	−0.4	+1.6	+1.4	+0.8
42$^\circ$N	60$^\circ$N	−7.2	−10	−32	−3.8	−6.8	−37
	30$^\circ$N	−4.7	−6.6	−21	−4.9	−7.3	−23
	0$^\circ$	+0.2	−0.9	−7.4	+1.3	+0.7	−2.8
	30$^\circ$S	+5.1	+4.7	0.0	+1.8	+1.8	+0.6
	60$^\circ$S	−0.6	−0.1	−1.6	+2.0	+1.4	+0.6
14$^\circ$N	60$^\circ$N	−4.4	−6.8	−22	−1.1	−2.1	−16
	30$^\circ$N	−2.2	−3.8	−15	−0.8	−3.1	−16
	0$^\circ$	−0.7	−1.8	−12	−2.1	−3.8	−15
	30$^\circ$S	+4.7	+3.8	−3.8	+1.7	+1.0	−2.1
	60$^\circ$S	−1.3	−2.0	−4.6	+0.4	−0.3	−3.5
1$^\circ$S	60$^\circ$N	−0.9	−2.0	−8.5	+2.1	+0.8	−3.6
	30$^\circ$N	+1.1	0.0	−6.6	+2.6	+1.5	−8.3
	0$^\circ$	−0.5	−2.4	−14	−2.2	−3.9	−17
	30$^\circ$S	+1.9	+0.1	−12	−0.7	−1.9	−9.8
	60$^\circ$S	−4.1	−4.7	−9.7	−3.9	−4.5	−13
17$^\circ$S	60$^\circ$N	+2.2	+1.7	−0.7	+3.5	+3.2	0.0
	30$^\circ$N	+3.3	+2.9	+0.2	+3.7	+3.1	−1.7
	0$^\circ$	+0.3	−1.1	−9.1	−0.8	−2.1	−10
	30$^\circ$S	−5.2	−7.5	−24	−5.0	−6.9	−20
	60$^\circ$S	−5.1	−9.1	−31	−8.2	−10	−28
50$^\circ$S	60$^\circ$N	+2.7	+2.5	+1.4	+2.2	+2.7	+0.4
	30$^\circ$N	+2.4	+2.2	+1.4	+2.7	+2.7	−0.7
	0$^\circ$	+1.3	+0.6	−2.7	+0.5	−0.6	−5.2
	30$^\circ$S	−7.7	−10	−27	−6.6	−8.6	−21
	60$^\circ$S	−6.5	−12	−49	−9.9	−13	−31

Ozone zonal mean column response in percent at specific latitudes 5 months after the eruption for all modelled scenarios. Latitudes shown highlight the impact on the tropical (0$^\circ$), mid- (30$^\circ$N and 30$^\circ$S), and high- (60$^\circ$N and 60$^\circ$S) latitudes.

The locations of 314 stratovolcanoes that have erupted during the Holocene in the American cordilleras are indicated by triangles in Fig. 1^62^. Six volcanoes were selected as proxies to investigate the latitudinal dependence of the impact of a large, explosive volcanic eruption on stratospheric ozone and are indicated in black. These specific volcanoes have all erupted with an eruption of VEI 6 or greater in the Holocene era and were chosen on the basis of their coordinates to cover a wide spread of latitudes for informational and not prognostic purposes. These locations are: Mt. Katmai (58$^\circ$N, 155$^\circ$W; Alaska, USA), Mt. Mazama (42$^\circ$N, 122$^\circ$W; Oregon, USA), Mt. Ilopango (14$^\circ$N, 89$^\circ$W; El Salvador), Mt. Quilotoa (1$^\circ$S, 79$^\circ$W; Ecuador), Mt. Huaynaputina (17$^\circ$S, 70$^\circ$W; Peru), and Mt. Aguilera (50$^\circ$S, 74$^\circ$W; Chile). We investigate the impact of the seasonality and halogen content of explosive volcanic eruptions at the latitude of each of these volcanoes for eruptions occurring on January 25, 2025 and July 25, 2025. Each evaluation is terminated at the end of year 2032.

The Shared Socioeconomic Pathways (SSP) scenario 3–70 chemical emissions boundary conditions were selected for all evaluations of eruptions reported in this work. The labelled scenario 3-70 is a combination of the SSP3 and Representative Concentration Pathways (RCP) 7.0 scenarios: SSP3 is defined with a fuel-use scenario that is close to that of the present day, and RCP7.0 is a scenario with 7.0 W m$^\text {-2}$ radiative forcing at the end of the year 2100^63^. For the metrics controlling stratospheric ozone response to volcanic eruption (e.g., vertical profile and magnitude of temperature of air, specific humidity, aerosol and trace gas emissions), the various SSP scenarios are not significantly different in the years 2025–2032. As a consequence, the selection of one SSP scenario over another is unlikely to bias the outcome of the simulated scenarios.

## Results and discussion

Volcanic eruptions were simulated in both January and July 2025, but for brevity we primarily present the results of volcanic eruptions occurring in July, except when comparing differences between the seasonal conditions. Likewise, unless otherwise noted, we typically present a sub-selection of the volcanic latitude scenarios considered. Additional scenarios are included in the Supplementary material as highlighted in the text.

### Evolution of the volcanic aerosol perturbation

Figure 2 illustrates the evolution of the stratospheric aerosol column as surface area density (SAD, $\mu$m$^2$/cm$^3$) for three representative volcanic scenarios with a stratospheric injection of 5 Tg SO$_\text {2}$ versus the quiescent scenario. The colorbar at the top of the figure indicates the difference in the amount of column aerosol in the volcanic eruption scenario versus the quiescent scenario; note that the contours are not scaled in linear increments. The date (July 25, 2025) and location of each volcanic eruption are indicated with a green triangle and a dashed line. Three latitudes of injection are shown in order to demonstrate a range of aerosol transport and evolution: (a) 42$^\circ$N, (b) 1$^\circ$S, and (c) 50$^\circ$S. Figures S1 and S2 in the Supplementary material display the SAD for all six latitudes studied for eruption scenarios in July and January 2025. In all three eruption scenarios in Fig. 2, the aerosol perturbation due to the eruption persists in significant quantities for approximately 2–2.5 years. The maximal increase in aerosol SAD is seen around the location of each eruption 2–5 months after the eruption. The background SAD is on the order of 1–5 ${\mu }$m$^\text {2}$/cm$^\text {3}$, meaning the increases due to the volcanic eruptions shown here are elevated by up to approximately a factor of 25. At the peak of the SAD values, the standard deviation for all model ensembles is about 35% (including Figs. S1 and S2), indicating the variation of model ensemble members. The summer mid-latitude Northern Hemisphere (NH) eruption, (a), produces an aerosol veil that remains almost entirely confined to the NH, with a maximum perturbation of 15–19 $\mu$m$^2$/cm$^3$ persisting 5 months for all latitudes above 45$^\circ$N. The tropical eruption, (b), produces an evenly distributed global aerosol veil. Though the maximal increase in SAD is only about half that of the SAD enhancements in (a) and (c), the perturbation persists significantly longer than either higher latitude scenario as a consequence of entrainment of aerosol into the deep branches of the Brewer–Dobson pattern. The winter mid-latitude Southern Hemisphere (SH) eruption, (c), is substantially similar to the summer mid-latitude NH eruption, excepting the enhanced inter-hemispheric transport beginning around October 2025. This effect is partially a consequence of the fact that the volcanic injection for the winter eruption occurred deeper in the stratosphere due to the lower winter tropopause. These scenarios did not differ qualitatively from other mid-latitude eruptions of the same seasonality as in panels (a) and (c). Considering that we maintained a constant injection vertical profile between all experiments, this shows that the main factor describing the spread of the volcanic aerosol is the latitude of the eruption, consistent with recent work^43^.

The persistence of the aerosols is governed by their atmospheric lifetimes, which can be described by their e-folding decay time, i.e., the time it takes for the amount of aerosols to decrease by a factor of *e*. The average global e-folding decay time of the stratospheric sulfate aerosol burden from a volcanic eruption was computed using the method of Marshall et al.^40^ and is provided in Fig. 3 for the different injection latitudes and injection scenarios: Sulfur dioxide only, indicated with black and blue symbols and lines for the July and January 2025 eruption scenarios, respectively, and high co-injected halogens for the July 2025 eruption scenario indicated by red symbols and lines. Bars indicate the standard deviation of the stratospheric aerosol burden across the model ensembles for each eruption scenario. Dependence on latitude is observed for all eruption scenarios; however, no significant dependence within ensemble uncertainty is observed for the type of injection (SO$_\text {2}$ only or with high co-injected halogens) or season. The average global e-folding decay times for the stratospheric aerosol burden for the scenarios shown in Fig. 2 panels (a)–(c) are 5.5, 11.3, and 6.3 months, respectively. The tropical eruption in panel (b) has the longest average global e-folding decay time, as the aerosol veil is more persistent than for eruptions occurring at other latitudes. Average global e-folding decay times for the stratospheric aerosols for all the eruption scenarios studied (differences in season, latitude, and halogen loading) range between 4.6–11.5 months. The larger magnitude average global e-folding decay times (above 7 months: 7.7–11.5 months) correspond to eruptions occurring in the tropical latitudes between about 20$^\circ$N–20$^\circ$S, while average global e-folding decay times for extra-tropical eruptions are between 4.6–6.4 months in the NH and SH. These decay times are similar to the e-folding decay times of 4–12 months reported in Marshall et al.^40^, who also found latitude to be the most important factor for average global e-folding decay time but did not consider seasonality or halogen injection, as was done here.

Some difference is observed in the average global e-folding decay times for extra-tropical eruptions occurring in January vs. July, with the January (July) eruption leading to a slightly more persistent stratospheric aerosol layer in the NH (SH). The difference in Brewer–Dobson circulation and height of the tropopause during the winter in both hemispheres is a likely explanation for these differences. However, the spread of the standard deviation of the model ensembles is large, so a seasonal impact on average global e-folding decay time is only suggested. When increased stratospheric aerosol is present in the atmosphere, it offers additional surfaces for heterogeneous chemistry to occur. This is evidenced by the changes in different chemical species in the atmospheric column, such as nitrogen and halogen compounds, which in turn will affect ozone chemistry.

### Ozone response to volcanic sulfate aerosol perturbation

The volcanic aerosol perturbation to heterogeneous chemistry and to dynamics of the chemistry-climate system leads to changes in the ozone layer following the stratospheric injection of SO$_\text {2}$ from an explosive volcanic eruption. Figure 4 provides the percent difference in total column ozone between a volcanic eruption on July 25, 2025 featuring a 5 Tg SO$_\text {2}$ injection to the stratosphere and the baseline control as a function of latitude and time. Figure S3 in the Supplementary material displays the ozone change following an eruption on January 25, 2025. Panels (a)–(f) depict eruptions occurring at progressively more southern locations (58$^\circ$N–50$^\circ$S), with the latitude and date of the volcanic eruption depicted as a green triangle and a dashed line. Red and blue coloration indicates a thinning or thickening of the ozone layer, respectively, as qualified by the colorbar at the top of the figure, noting that the scale does not increase linearly. As shown in the main (bottom left) subpanels, ozone decreases are observed regionally after the eruption with different extent and magnitude depending on eruption latitude, and ozone increases are observed in the opposite hemisphere of the eruption followed by ozone losses typically starting 0.5–1 year after the eruption. The top left subpanel of each figure panel demonstrates the global-average ozone change relative to the control experiment as a function of time for the volcanic scenario, shown as a black line. Ensemble 2$\sigma$ standard deviation in column ozone change is shown for each volcanic eruption scenario in green shading. The largest deviations are seen at the time of the austral springtime ozone depletion in all years. The bottom right subpanel provides mean temporal ozone change as a function of latitude. Here, the black line represents the time-averaged ozone column change versus latitude. Ensemble standard deviations are shaded as in the top left subpanel, highlighting the largest deviations in the high-latitude regions. Extra-polar hemispheric (0$^\circ$–80$^\circ$N and 0$^\circ$–80$^\circ$S) and global mean (80$^\circ$S–80$^\circ$N) percent changes in column ozone are presented in the top right corner and represent latitudinal and temporal averages of the ozone changes for the first 3 years following the eruption as displayed in the main (bottom left) subpanels of (a)–(f). The percentages indicate the average impact on ozone as observed on both a regional (NH and SH) and a global scale.

All SO$_\text {2}$ only eruption scenarios considered in Fig. 4 result in ozone depletion. Note the greater regional impacts on ozone by the extra-tropical eruptions at higher latitudes in both hemispheres compared to the tropical eruptions in the region of the eruption. EESC remains significantly elevated in the year 2025 relative to the natural halogen background. As a consequence of this elevated EESC, heterogeneous suppression of NO$_{\rm x}$ chemistry via N$_2$O$_5$ hydrolysis leads to increases in reactive halogen species. In the gas phase, the NO$_\text {2}$ + O reaction rate is elevated in regions of high ozone depletion. Where increases in ozone layer thickness occur, this reaction rate decreases relative to the baseline control scenario. Partitioning of halogen reservoirs to reactive chlorine (Cl$_\text {x}$=Cl+ClO) and reactive bromine (Br$_\text {x}$=Br+BrO) species increases their concentrations by up to 10% depending on the latitude of eruption. Additionally, we find that reactive halogen enhancements are more persistent after a tropical eruption than after eruptions at higher latitudes, in line with the trend of average global e-folding decay time vs latitude of eruption. Klobas et al.^35^ demonstrated, using a 2D chemical-transport-aerosol model, that heterogeneous chemical reactions following large, explosive tropical eruptions lead to reductions in the thickness of the ozone layer for time periods when EESC levels are elevated. Our results support their findings, though we find moderately lower magnitude global-temporal average ozone losses for the present day than reported by Klobas et al.^35^, because the SOCOL-AERv2 model used in the current work accounts for both radiative-dynamical and chemical changes to column ozone instead of chemical changes only.

The global impact of extra-tropical volcanic eruptions on stratospheric ozone is attenuated relative to tropical eruptions, but these eruptions tend to disproportionately decrease ozone in the hemisphere in which they occur, particularly during the springtime following the eruption. Tropical eruptions increase the magnitude of polar ozone depletion events in both hemispheres for several years following the eruption. After approximately 3 years, as the stratospheric aerosol burden recovers to its quiescent value (depicted in Fig. 2), volcanic ensemble ozone variation is indistinguishable from variation in the baseline scenario. For eruptions at all latitudes, an increase in ozone in the opposite hemisphere to the eruption is observed for the initial 0.5–1 year after the eruption, depending on latitude of eruption. After the initial increase in ozone in the opposite hemisphere to the eruption, thinning of column ozone is observed from the polar regions to approximately 30$^\circ$N/S of varying duration between 0.5–2.5 years, increasing the austral/boreal springtime ozone depletion events as well as contributing to a regional ozone decrease. This regional decrease in the NH following an eruption in the SH is stronger than the SH decreases after a NH eruption (e.g., comparing panels (a) and (f)). This indicates a dependence on the season of eruption, with a winter eruption leading to more persistent ozone depletion following the period of ozone increase in the opposite hemisphere to the eruption. The persistence of this ozone depletion is reversed for the January eruption scenarios for the two latitudes, again with the winter eruption giving a more persistent opposite hemisphere depletion.

The initial ozone asymmetry observed here is consistent with the observations following the Mt. Pinatubo eruption in 1991 (15$^\circ$N), which erupted at a similar latitude to the location of the eruption in panel (c) and was argued to be caused by a combination of chemistry and dynamics. Poberaj et al.^21^ and Aquila et al.^5^ found that changes in dynamics combined with aerosol heating following the Mt. Pinatubo eruption led to an increase in Brewer–Dobson circulation with enhanced tropical upwelling and extra-tropical downwelling in the SH, increasing the transport of ozone from the tropics to SH mid-latitudes for a year after the eruption. Dhomse et al.^22^ discussed the description of this asymmetry by different models and came to a similar conclusion as the studies by Poberaj et al. and Aquila et al.^5,21^. A delayed ozone decrease in the SH was observed to begin towards the end of 1991 after the initial increase; however, comparison of the longer-term impact on ozone after the Mt. Pinatubo eruption from observations of the SH stratosphere is difficult given the VEI 5 eruption of Cerro Hudson (46$^\circ$S) on August 12–14, 1991, influencing the atmospheric composition^5,9^.

To explore the impact of dynamics further, Fig. 5 shows the model output variable, ’ideal age’, which describes the age of air, where the ideal age of an air parcel is 0 at the Earth’s surface and one day is added per model day. Gridpoint values indicate the integrated column average ideal age of air of all parcels mixed into that particular gridpoint location. Changes in ideal age of air correlate with transport in the model. Increases (blue coloration) and decreases (red coloration) are described by the colorbar at the top of the figure. The difference in ideal age of air between a sulfur dioxide only volcanic eruption and the baseline control is shown as a function of latitude and time for an eruption in the NH, panel (a) 42$^\circ$N, and the SH, panel (b) 50$^\circ$S. The date and location of the volcanic eruption is highlighted with a green triangle and a dashed line. The scenarios were chosen to represent the changes in the NH and SH, respectively. Increases in ideal age of air (shown in blue) in a specific location denote older air being transported into the location or static air remaining at the location, and decreases (shown in red) indicate the opposite when comparing the change in ideal age of air between the volcanic eruption scenario and the baseline scenario. Comparison of Fig. 4 panels (b) and (f) with Fig. 5 panels (a) and (b) shows that the increases in ozone observed in Fig. 4 approximately correspond to the increases in ideal age of air (“old air”) for eruptions in both the NH and SH, whereas the ozone depletion areas approximately correspond to decreases in the ideal age of air (“young air”). That the asymmetry seen in Fig. 4 for ozone is observed for the ideal age of air points to a change in dynamics following the volcanic eruptions. This is further evidenced by increased water vapor in the tropics and stratospheric heating in the model outputs. For the tropical eruptions, the global surface temperature in the model outputs decrease after the volcanic eruption by approximately 0.7–1.1 K for all modelled scenarios, in agreement with observations following eruptions of similar magnitudes confirming that the climate and meteorology in the model is responding to a volcanic eruption in a manner comparable to observational data^9,64–66^. The extra-tropical eruptions result in a global surface temperature decrease of 0.4–1.0 K, with the largest changes being observed after a winter eruption, seen in both hemispheres.

### Ozone response to halogen-rich volcanic eruptions

Here we consider the potential stratospheric impact of inorganic halogen co-injection from an explosive volcanic eruption on ozone. Figure 6 shows changes in percent of total column ozone for sulfur dioxide only eruptions, along with intermediate and high co-injected halogen scenarios versus the volcanic quiescent baseline scenario as a function of latitude and time. The left column shows SO$_\text {2}$ only (5 Tg SO$_\text {2}$) as shown in panels (b), (d), and (f) of Fig. 4, the middle column shows intermediate co-injected halogen (5 Tg SO$_\text {2}$, 0.05 Tg HCl, 0.0005 Tg HBr), and the right column shows high co-injected halogen (5 Tg SO$_\text {2}$, 0.5 Tg HCl and 0.005 Tg HBr). Three latitudes of injection are shown: (a)–(c) 42$^\circ$N, (d)–(f) 1$^\circ$S, and (g)–(i) 50$^\circ$S. Latitude and date (July 25, 2025) of the volcanic eruptions are indicated by a green triangle and dashed line. Similar to Fig. 4, the main bottom left panels indicate a thickening of the ozone layer in blue, and here a thinning of the ozone layer is indicated in tones of yellow, orange, red, and purple. Note that the scale is not incremented linearly. Like Fig. 4, the top left- and bottom right subpanels show the ensemble global column average deviations versus time (top left) and regio-temporal column average deviations versus latitude (bottom right), respectively. The green shading in the subpanels indicates the ensemble standard deviations (2$\sigma$) for the volcanic eruption. Extra-polar hemispheric (0$^\circ$–80$^\circ$N and 0$^\circ$–80$^\circ$S) and global mean (80$^\circ$S–80$^\circ$N) column ozone changes are presented in the top right corner panel for the first 3 years following the eruption.

With increasing quantities of co-injected halogen at constant SO$_\text {2}$, regional ozone depletion is amplified, with peak ozone losses increasing from $-25\%$ up to $-50\%$; note the scale of the colorbar in Fig. 6 compared to that in Fig. 4. Ozone losses also persist longer, from about 2.5 years to more than 4 years. The thinning of the ozone column spreads further with the high-halogen injection scenario compared to the other two types of injections, showing substantial global ozone depletion for all latitudes of eruption. The annual boreal/austral springtime ozone depletion following the eruption increases with halogen injection quantities. As the amounts of the volcanic halogen gas increase, the chemical character of ozone depletion becomes increasingly dominant. This effect is manifest by fewer enhancements in ozone in the opposite hemisphere as a consequence of dynamical perturbation. For the tropical eruption in panel (f), the ozone depletion is spread across the globe, increasing the strength of the annual boreal/austral springtime ozone depletion events for a few years after the volcanic eruption. The mid-latitude eruptions increase the springtime ozone depletion substantially for the first year after the eruption, and the high-halogen injections have a widespread regional ozone thinning effect beyond the springtime ozone depletion events. We note that a high co-injection of halogen eruption in the SH leads to ozone depletion in the NH of the same magnitude as an intermediate halogen injection in the NH (comparing regional averages for the NH in panels (b) and (i)). This demonstrates that the increase in halogen content reaching the stratosphere from an eruption can significantly change ozone on a global level, even for higher latitude eruptions when the halogen content is sufficiently large. The impact on zonal mean column ozone for all modelled scenarios is collated in Table 1, showing the percent change in ozone 5 months after the eruption (at the approximate peak loss of ozone) for selected latitudes: 60$^\circ$N, 30$^\circ$N, 0$^\circ$, 30$^\circ$S, and 60$^\circ$S. This highlights the immediate latitudinal ozone impact following an explosive volcanic eruption.

Our simulations show that large, explosive eruptions with stratospheric injections of 0.05–0.5 Tg HCl and 0.0005–0.005 Tg HBr, regardless of their latitude, could have a significant impact on ozone globally. A recent study by Staunton-Sykes et al.^46^ investigated the impact of co-injecting halogens with SO$_\text {2}$ for a contemporary volcanic eruption at a single latitude only and also found large ozone losses, but because they used injections of 2–11 times more SO$_\text {2}$ than in the present work as well as 3–30 times higher halogen content than our high-halogen injection scenario, further quantitative comparison is difficult. Klobas et al.^35^ also previously showed large ozone losses from a contemporary explosive volcanic eruption with co-injected HCl using a 2D model for a single latitude and season. Many volcanoes worldwide have the capability to produce eruption scenarios as described in the present study in the near future, with the typical recurrence rate of VEI 6–7 eruptions being 80 and 500 years, respectively^67–71^. Regionally, a detrimental impact on human health could be anticipated for the high-halogen injection scenario investigated here, where the subsequent increase in UVB radiation could be dramatic^39,46^. It is important to note that, if occurring at a high latitude, even smaller-scale explosive eruptions have the potential to transport significant amounts of halogens into the stratosphere, since the tropopause is lower at high latitudes than in the tropics. For instance, measurements in the plume of Mt. Hekla, Iceland (63$^\circ$N), 35 hours after its VEI 3 eruption in 2000 showed that as much as 75% of the erupted volcanic HCl was transported to the lower stratosphere^72,73^. If persistent trends in glacial melting continue as the climate evolves, surface loading over ice-covered volcanoes at high latitudes will decrease, possibly resulting in a greater frequency of high-latitude eruptions than in the present time^74^.

### Impact of seasonality on ozone response to volcanic eruptions

Does the time of year of an explosive volcanic eruption impact the extent of stratospheric ozone loss? Figure 7 shows changes in ozone averaged over 3 years following a volcanic eruption as a function of the season in which the eruption occurred. The modelled output has been split to match the seasons of the NH and SH for each of the six eruption latitudes as follows, January 2025: NH winter, SH summer and July 2025: NH summer, SH winter. The NH averages are shown in blue, SH in red, and global changes in grey. Summer eruptions are shown in the left column (panels (a), (d), and (g)), winter eruptions in the middle column (panels (b), (e), and (h)) and the difference in ozone depletion between the two seasons (summer – winter) is shown in the right column (panels (c), (f), and (i)). The rows indicate the type of injection: SO$_\text {2}$ only, intermediate co-injected halogen, and high co-injected halogen for the top, middle, and bottom rows, respectively. The latitudes of eruptions are labelled V1–V6, spanning the range of latitudes studied here from north to south (58$^\circ$N–50$^\circ$S). Uncertainty whiskers are plotted in black (1$\sigma$).

Highlighting the ozone losses for the two seasons (comparing panels (a), (b), (d), and (e) with panels (g) and (h)) in Fig. 7, the ozone losses in the high-halogen co-injection scenarios are much more dramatic than the intermediate halogen or SO$_\text {2}$ only injection scenarios regardless of season. In panels (c), (f), and (i), however, it is apparent that in all cases the differences between the seasons (summer – winter) are small on a 3-year timescale, with uncertainty whiskers transiting the 0% ozone change mark in most scenarios. Seasonal differences range between $-0.6\%$ and +1.0% for the intermediate halogen and SO$_\text {2}$ only injection scenarios and $-0.6\%$ and +1.8% for the high-halogen co-injection scenario. The largest seasonal differences are observed for the two northernmost eruptions (58$^\circ$N and 42$^\circ$N; V1 and V2) in the high-halogen co-injection scenario (panel (i)) and the northernmost eruption (58$^\circ$N; V1) for the intermediate halogen and SO$_\text {2}$ only injection scenarios. These differences indicate a stronger ozone depletion following a winter eruption.

While the differences shown in Fig. 7 appear to indicate that stratospheric ozone is not strongly dependent on the season of occurrence of an explosive volcanic eruption, it is important to highlight that these results are for a 3-year time average. Looking in more detail as a function of time, significant ozone differences become apparent depending on the season of the eruption. In Fig. 4, the initial ozone increase in the hemisphere opposite the eruption is larger for the winter eruptions than for the summer eruptions (e.g., comparing the SH eruptions in panels (f) and (e) with the NH eruptions in panels (a), (b), and (c)). Moreover, in Figs. 4 and 6, there is a seasonal dependence on the persistence of the delayed ozone depletion (after the initial ozone increase) in the opposite hemisphere to the location of the volcanic eruption, for which ozone losses increase for a winter eruption compared to a summer eruption (e.g., comparing the NH ozone depletion poleward of 45$^\circ$N in years 2027–2029 in panel (f) of Fig. 4 with the SH ozone depletion poleward of 45$^\circ$S for those same years in panel (a)). These two observations are also seen, when comparing Fig. 4 with Fig. S3 in the Supplementary material. This increased magnitude and persistence of the ozone depletion in the opposite hemisphere to the eruption is seen in all modelled scenarios, regardless of halogen content. With increasing halogen content co-injected with SO$_\text {2}$, the extent of this ozone depletion reaches closer to the equator.

The extent of the global spread of the formed aerosols depends on the season of the eruption as well as the latitude. For the higher latitude eruption scenarios, the aerosols are more confined to the hemisphere of the eruption following a summer eruption, whereas the aerosols are generally distributed globally faster (within 10 months) after winter eruptions in both hemispheres for the extra-tropical eruptions investigated here (See Figs. S1 and S2 in the Supplementary material). This could in part be due to the lower tropopause in the winter, as the injection of SO$_\text {2}$ and halogen species are kept at the same injection height for all modelled scenarios. As discussed above, Fig. 3 shows no significant dependence of the average global aerosol e-folding decay time on season within the spread of model ensembles. The global surface temperature shows a seasonal dependence for extra-tropical eruptions, with an additional decrease of 0.1–0.3 K in the winter compared to the summer for the eruptions at 58$^\circ$N, 42$^\circ$N, and 50$^\circ$S. No seasonal dependence on the temperature was observed following the tropical eruption scenarios, with less than 0.1 K difference between summer and winter eruptions. The largest differences in temperature are observed for the high-halogen co-injection scenarios, as is the case for the seasonal ozone impact.

This work provides the first comprehensive study specifically focusing on the stratospheric ozone impact of the latitude and seasonality of volcanic eruptions. Using the results to return to the June 15, 1991 Mt. Pinatubo eruption, a few comments on a hypothetical change of eruption seasonality can be made. Given the tropical latitude of the Mt. Pinatubo eruption (15$^\circ$N), had it occurred in the winter rather than the summer, there may have been some differences in ozone as a function of time particularly in the SH, but the overall impact of the eruption on stratospheric ozone would likely not have been significantly different. We base this statement on the 3-year average ozone depletion data presented in panel (c) of Fig. 7. The change in 3-year average ozone depletion between winter and summer eruptions for the volcano labeled V3 (14$^\circ$N) has an error transiting the 0% ozone change line, indicating that no statistical difference can be inferred from the experimental treatment. However, the Mt. Pinatubo eruption occurred coincident with the regional transit of tropical cyclone Yunya, which significantly enhanced ambient water vapor^9,10^ and improved the gas scrubbing efficiency of the volcanic plume^13^. A different impact of seasonality on ozone may have been possible if the eruption had happened in the winter, as the Western North Pacific typhoon season terminates in November^75^. Had a winter eruption of Mt. Pinatubo into a less humid atmosphere resulted in a reduced gas scrubbing efficiency of the plume, the overall impact on ozone could have been amplified. Using the estimated stratospheric SO$_\text {2}$ injection from the Mt. Pinatubo eruption of 14–23 Tg SO$_\text {2}$^4–7^ and HCl:SO$_\text {2}$ ratio of 0.4^8,46^ the total enhancement of stratospheric HCl would be significant. If 5% of the Pinatubo HCl had reached the stratosphere, it would have been equal to 0.3–0.5 Tg of HCl with an impact on ozone comparable to the high-halogen injection scenario presented here.

## Summary

This work presents a systematic large-ensemble modelling study of the sensitivity of stratospheric ozone to the latitude, season, and amount of halogen injected into the stratosphere by explosive volcanic eruptions. Our results show that both aerosols and ozone have a strong dependence on eruption latitude for all injection scenarios investigated. Higher latitude (NH and SH) eruptions tend to produce a large regional-to-hemispheric impact on ozone and a smaller global impact, whereas tropical eruptions tend to exert an ozone impact more global in scale. Changes in total column ozone and aerosol SAD return to the baseline ensemble variation approximately 2.5–3 years after the eruption regardless of latitude or season for the SO$_\text {2}$ only injection scenario; however, this time increases with co-injected halogen species. Increasing the amount of halogen co-injected with SO$_\text {2}$ in the eruption column increases the degree of ozone depletion and its extent and duration. Importantly, higher latitude eruptions in the intermediate and high halogen co-injection scenarios also have a global impact on ozone, with a global ozone depletion lasting for more than 4 years.

In all the injection scenarios investigated here, the impact of a volcanic eruption on stratospheric ozone has some dependence on seasonality of the eruption for the extra-tropical eruptions, with a winter eruption resulting in ozone depletion in the opposite hemisphere (poles to approximately 30$^\circ$N/S) to the eruption that is more persistent than that following a summer eruption. This opposite hemisphere ozone depletion follows an initial increase in ozone, which is larger for winter eruptions than summer eruptions. Interestingly, these two effects, larger increases in stratospheric ozone in the opposite hemisphere of eruption followed by more persistent decreases, tend to cancel out in multi-year averages of ozone loss, providing an overly simplistic appearance of insignificant seasonal influence of the volcanic eruption.

The halogen loading of the eruption has an insignificant impact on the aerosol formation, which is expected as the aerosol formation is dependent mostly on the injected SO$_\text {2}$. An increasing amount of volcanic halogen co-injected into the stratosphere with SO$_\text {2}$ leads to further thinning of ozone. For the high-halogen co-injection scenarios investigated here, the ozone losses are severe: 6–10% globally and 10–14% hemispherically on a 3-year time scale, with peak ozone losses exceeding 20% on a global scale in the first year following the volcanic eruption.

## Supplementary Information

Supplementary Information.

## Data Availability

The post-processing and visualisation of the data were performed with CDO and Python. Post-processed data are available upon request from the corresponding authors.
