# Supplementary material for: Sensitivity of stratospheric ozone to the latitude, season, and halogen content of a contemporary explosive volcanic eruption
Source: Sci Rep. 2023 Apr 20;13:6457. doi: 10.1038/s41598-023-32574-9 (PMC10119174; doi:10.1038/s41598-023-32574-9)
Supplement: Supplementary file 1 — Supplementary Information. [file 41598_2023_32574_MOESM1_ESM.pdf]

Supplementary Material for Sensitivity of Stratospheric Ozone to the Latitude, Season, and Halogen Content of a Contemporary Explosive Volcanic Eruption

Freja F. Østerstrøm*, **J. Eric Klobas***, **Robert P. Kennedy** , **Anita Cadoux** , and **David M. Wilmouth**

*foesterstroem@huarp.harvard.edu
*klobas@huarp.harvard.edu

Overview of Supplementary Figures

Aerosol and ozone responses to 5 Tg SO_2 only eruption scenarios:

- Figure S1: Aerosol SAD for eruption scenarios on July 25, 2025 for all six latitudes (58°N to 50°S).
- Figure S2: Aerosol SAD for eruption scenarios on January 25, 2025 for all six latitudes (58°N to 50°S).
- Figure S3: Ozone response to a volcanic eruption on January 25, 2025 for all six latitudes (58°N to 50°S).

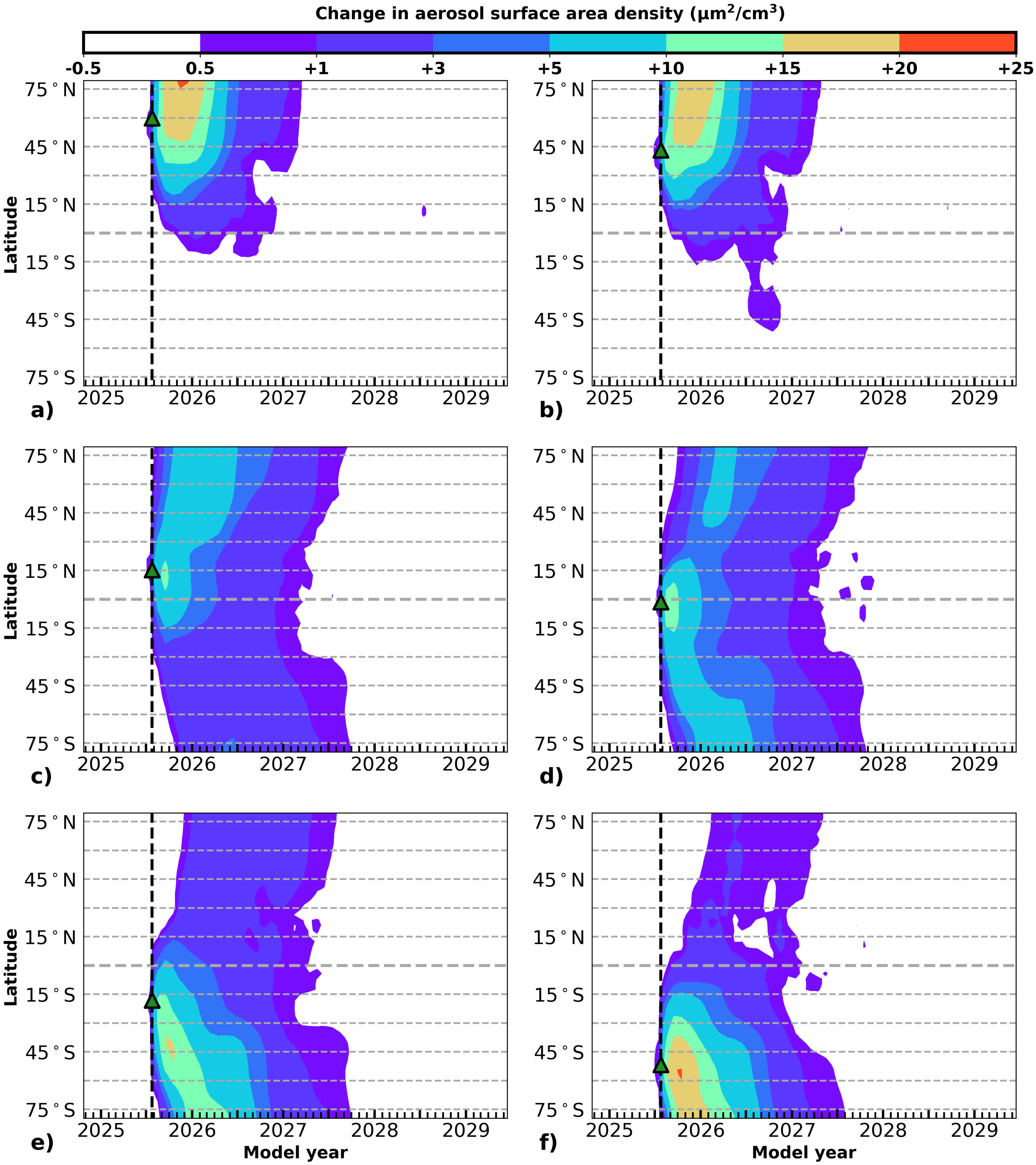

Figure S1. Aerosol surface area density (SAD) response to 5 Tg SO_2 injection (SSP3-70 July 25, 2025) as a function of latitude. In all panels, computed metrics reflect the difference in SAD between the volcanic eruption perturbation model ensemble and the baseline control model ensemble shown for a 4-year time-horizon. Total column response of aerosol SAD is indicated by the colorbar. The latitudes of eruption are: **a)** 58°N, **b)**, 42°N, **c)** 14°N, **d)** 1°S, **e)**, 17°S, and **f)** 50°S. Latitude and date of each SO_2 injection are indicated by a green triangle and dashed line.

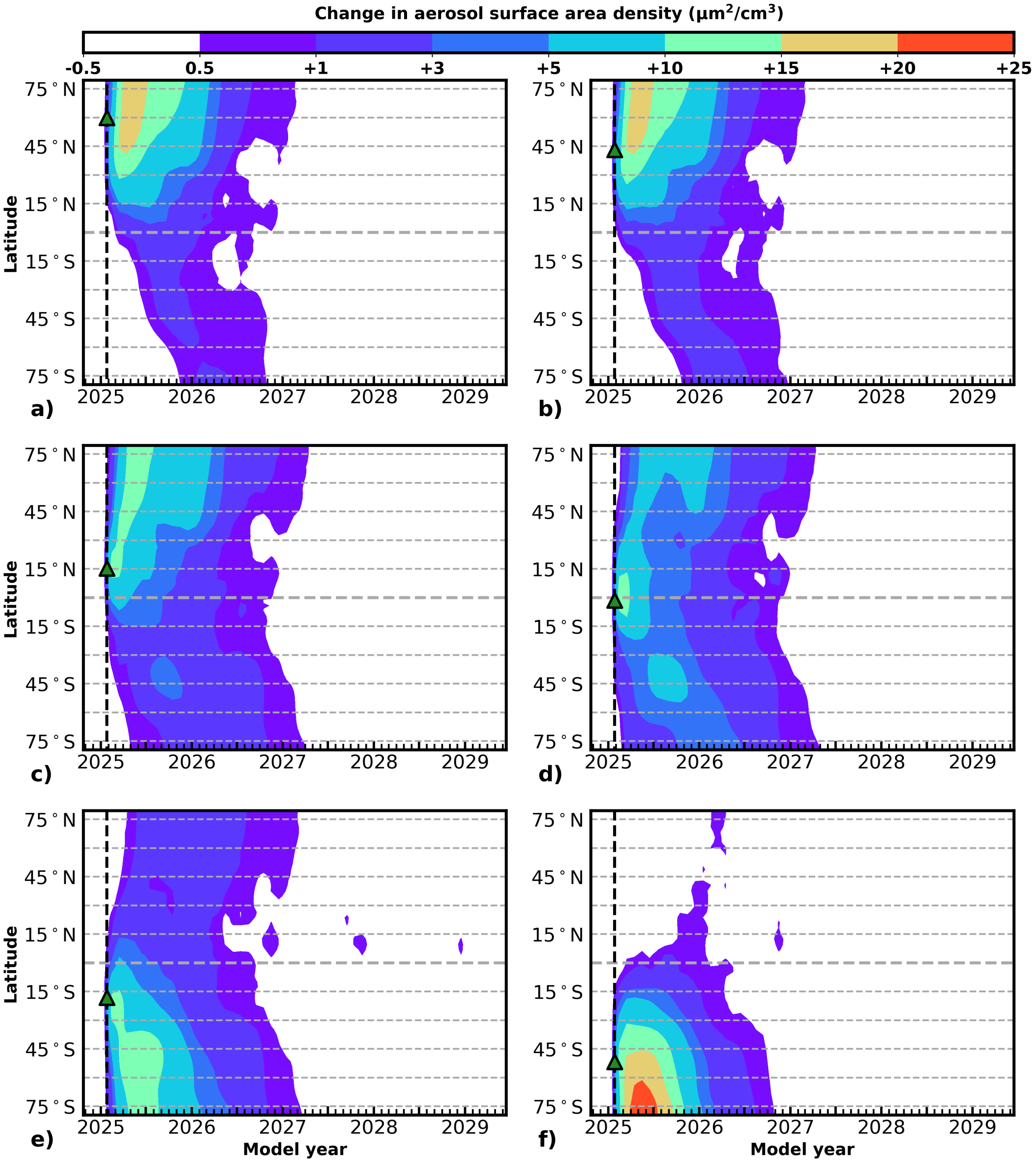

Figure S2. Aerosol surface area density (SAD) response to 5 Tg SO_2 injection (SSP3-70 January 25, 2025) as a function of latitude. In all panels, computed metrics reflect the difference in SAD between the volcanic eruption perturbation model ensemble and the baseline control model ensemble shown for a 4-year time-horizon. Total column response of aerosol SAD is indicated by the colorbar. The latitudes of eruption are: **a)** 58°N, **b)**, 42°N, **c)** 14°N, **d)** 1°S, **e)**, 17°S, and **f)** 50°S. The latitude and date of each SO_2 injection are indicated by a green triangle and dashed line.

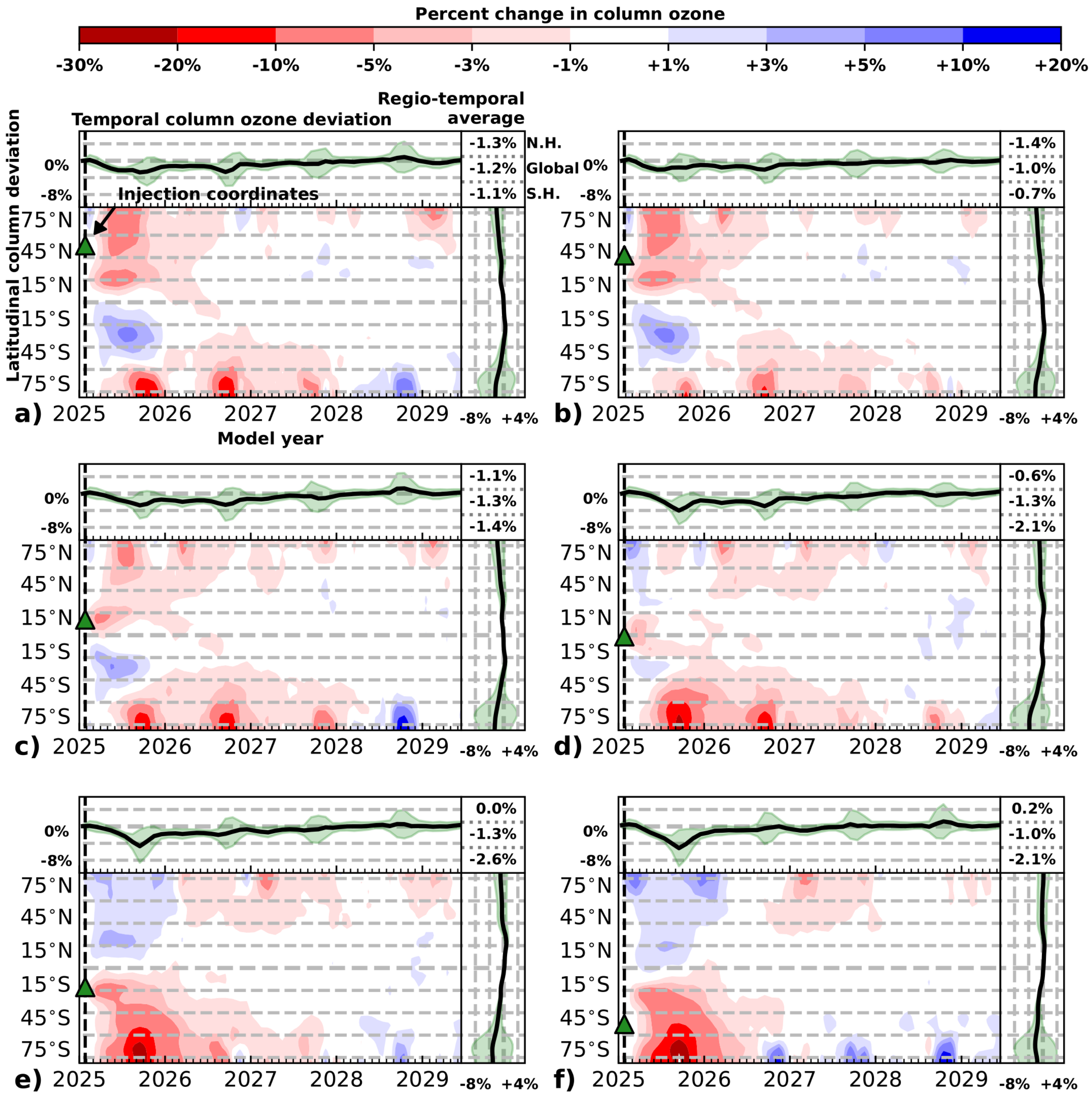

Figure S3. Ozone response to 5 Tg SO_2 injection (SSP3-70 January 25, 2025) as a function of latitude. The latitudes of eruption for panels **a)** through **f)** are: 58°N, 42°N, 14°N, 1°S, 17°S, and 50°S, respectively, with the latitude and date of SO_2 injection indicated by a green triangle and dashed line in each panel. In all graphical panels, computed metrics reflect the percent change in ozone between the volcanic eruption perturbation model ensemble and the baseline control ensemble shown for a 4-year time-horizon. Main panels (bottom left): Response of total column ozone in percent to the volcanic eruption as indicated in the colorbar (note that colorbar levels increment non-linearly). Top left panels: Global average response of total column ozone over time as indicated in the left scale. Bottom right panels: Response of total column ozone average over time versus latitude. Green shading in the top left and bottom right panels illustrates the variation (2σ) in the eruption perturbation ensemble; note that the increment between dashed lines in these panels is 4%. Top right corner panel: 3-year extra-polar hemispherical average (0°–80°N and 0°–80°S) and global average (80°S–80°N) of the total column ozone deviation, ordered as NH, Global, SH.
